# Construction and Validation of Novel Prediction Tools Based on Large Population-Based Database to Predict the Prognosis of Urachal Cancer After Surgery

**DOI:** 10.3389/fonc.2021.718691

**Published:** 2021-09-14

**Authors:** Xiaowen Yu, Chong Ma, Maoyu Wang, Yidie Ying, Zhensheng Zhang, Xing Ai, Linhui Wang, Shuxiong Zeng, Chuanliang Xu

**Affiliations:** ^1^Department of Urology, Changhai Hospital, Naval Medical University, Shanghai, China; ^2^Department of Geriatrics, Changhai Hospital, Naval Medical University, Shanghai, China; ^3^Senior Department of Urology, The Third Medical Center of PLA General Hospital, Beijing, China

**Keywords:** nomogram, predictors, prognosis, urachal cancer, SEER

## Abstract

**Background:**

Urachal cancer is a rare neoplasm in the urological system. To our knowledge, no published study has explored to establish a model for predicting the prognosis of urachal cancer. The present study aims to develop and validate nomograms for predicting the prognosis of urachal cancer based on clinicopathological parameters.

**Methods:**

Based on the data from the Surveillance, Epidemiology, and End Results database, 445 patients diagnosed with urachal cancer between 1975 and 2018 were identified as training and internal validation cohort; 84 patients diagnosed as urachal cancer from 2001 to 2020 in two medical centers were collected as external validation cohort. Nomograms were developed using a multivariate Cox proportional hazards regression analysis in the training cohort, and their performance was evaluated in terms of its discriminative ability, calibration, and clinical usefulness by statistical analysis.

**Results:**

Three nomograms based on tumor–node–metastasis (TNM), Sheldon and Mayo staging system were developed for predicting cancer-specific survival (CSS) of urachal cancer; these nomograms all showed similar calibration and discrimination ability. Further internal (c-index 0.78) and external (c-index 0.81) validation suggested that Sheldon model had superior discrimination and calibration ability in predicting CSS than the other two models. Moreover, we found that the Sheldon model was able to successfully classify patients into different risk of mortality both in internal and external validation cohorts. Decision curve analysis proved that the nomogram was clinically useful and applicable.

**Conclusions:**

The nomogram model with Sheldon staging system was recommended for predicting the prognosis of urachal cancer. The proposed nomograms have promising clinical applicability to help clinicians on individualized patient counseling, decision-making, and clinical trial designing.

## Introduction

Urachal cancer was first reported by Jacquin in 1863; it primarily occurs in men and constitutes <1% of bladder tumors (1, 2). Urachal cancer is a rare but highly malignant tumor arising from urachal remnant, which is located between the umbilicus and the dome of the bladder (1, 3). This hidden unusual location makes patients present symptoms such as hematuria and pain at advanced stages; about one-third of patients are already metastasized at diagnosis (4). Partial and radical cystectomy can be performed for nonmetastatic urachal cancer, both of which provide similar oncological outcome of 5-year survival rates ranging from 45% to 49% (2, 5). Partial cystectomy with *en bloc* excision of the urachal ligament, umbilicus, and dome of the bladder could achieve complete resection of tumor, fewer postoperative complication. and better quality of life compared with radical cystectomy; thus, it is most commonly recommended in clinical practice (6). The most common histological type of urachal cancer is adenocarcinomas, which account for nearly 90%, and other histological types such as urothelial carcinomas, squamous cell carcinomas, sarcomas, and undifferentiated carcinomas have also been reported (1, 2).

Due to the rarity of urachal cancer, there are no standardized protocols, and high level of evidence suggest that neoadjuvant, adjuvant, or salvage chemotherapy regimens confer survival benefit for recurrent and metastatic urachal cancer (4, 7). Nevertheless, adjuvant chemotherapy may be reasonable to treat patients at high risk of relapse (8). However, few studies have investigated prognostic factors that will help urologists and oncologists to predict prognosis and risk for relapse (9). Two staging systems are currently commonly used for urachal cancer: one was proposed by Sheldon et al. (10) in 1984, and the other simplified system was proposed by the Mayo Clinic in 2003 (11). Several studies have failed to find prognostic value using the Sheldon’s system or other parameters, such as tumor size, histological differentiation, immunohistochemical and serum markers, and this may also attribute to small size of study cohort (4, 12, 13).

A useful model is currently needed to be developed for stratifying patients with urachal cancer into different risks of prognosis; thus, patients with high risk would require more aggressive treatment and closer follow-up. The present study aims to develop nomograms for predicting the prognosis of urachal cancer based on clinicopathological parameters using the Surveillance, Epidemiology, and End Results (SEER) database and externally validate them using an independent cohort from two medical centers for potential clinical application.

## Methods

### Patient Selection

Training cohort data were collected from the SEER database of the National Cancer Institute (https://seer.cancer.gov) using SEER data that were accessed using the SEER*Stat version 8.3.9. We selected the database SEER Research Data, 9 and 18 Registries, which were submitted in November 2020 (1975–2018), representing approximately 30% of US population. Urachal cancer patients were identified by the International Classification of Diseases O-3 codes C67.7. Exclusion criteria were as follows (1): patients did not undergo surgery, or the surgery procedures were not available (2); patients with missing information on crucial covariates such as pathological information (TNM stage all absent) and vital status; and (3) survival time ≤1 months. Validation cohort of 45 patients and 39 patients diagnosed as urachal cancer were collected from Changhai Hospital and the Third Medical Center of PLA General Hospital from January 2001 to February 2021 in two high volume, tertiary care centers, respectively. The exclusion criteria were the same as mentioned previously. This retrospective study was designed in accordance with the ethical guidelines outlined in the Declaration of Helsinki and approved by the ethical boards of Changhai Hospital and the Third Medical Center of PLA General Hospital.

### Data Collection and Definition

Demographic and clinical variables were collected from SEER database. Pathological information that could not be defined precisely was referred as Gx, Tx, Nx, or Mx, while cases with blank pathological information in the database were excluded for analysis. We further restaged patients using the Sheldon and Mayo staging system. The criteria for Sheldon staging were as follows: patients with carcinoma *in situ* (CIS); Ta–1, N0, M0, localized disease were classified as Sheldon I; T2, N0, M0, localized disease were classified as Sheldon II; T3–4, N0, M0, regional disease were classified a Sheldon III; and T3–4 and/or N1 and/or M1, distant disease were classified a Sheldon IV. The criteria for Mayo staging were as follows: patients with CIS; Ta–2, N0, M0, localized disease were classified as Mayo I; T3–4, N0, M0, regional disease were classified as Mayo II; any T, N1, M0 reginal disease were classified as Mayo III; and any T, N2-3, and/or M1, distant disease were classified as Mayo IV. The corresponding demographic and clinical parameters of the validation cohort were retrospectively collected from medical records, and follow-up information was provided by patients or family members *via* telephone calls.

### Statistical Analysis

Continuous parametric data were compared using t-test, and categorical data were compared using the chi-square test. Kaplan–Meier method and log-rank test were used to compare cancer-specific survival (CSS) between groups. We randomly selected two-thirds of included cases from SEER database as training set, while the remaining as internal validation set. Univariable and multivariable Cox proportional hazards models were performed in training cohort. A backward step-down Wald selection method was applied to select independent risk factors (the entry and removal criteria were *p* < 0.05 and *p* < 0.10, respectively), and nomograms predicting the 3- and 5-year cancer CSS were developed using these independent risk factors. The performance of the nomogram was further validated using the internal and external validation cohort. The discrimination accuracy of nomogram was quantified using Harrell’s concordance index (c-index) (14, 15). Calibration curves were plotted to assess the calibration of the nomogram. Decision curve analysis (DCA) was performed to estimate the clinical usefulness of the nomogram by calculating the net benefits for a range of threshold probabilities (16). The X-tile software version 3.6.1 (Yale University, New Haven, CT, USA) was used to determine the optimal nomogram score cutoff value for classifying patients into different risk groups (17). Statistical significance *p* value was set at 0.05 with two sides. Statistical analyses were conducted using R software 4.0.4 (http://www.r-project.org).

## Results

### Patient Characteristics

**Figure 1:**
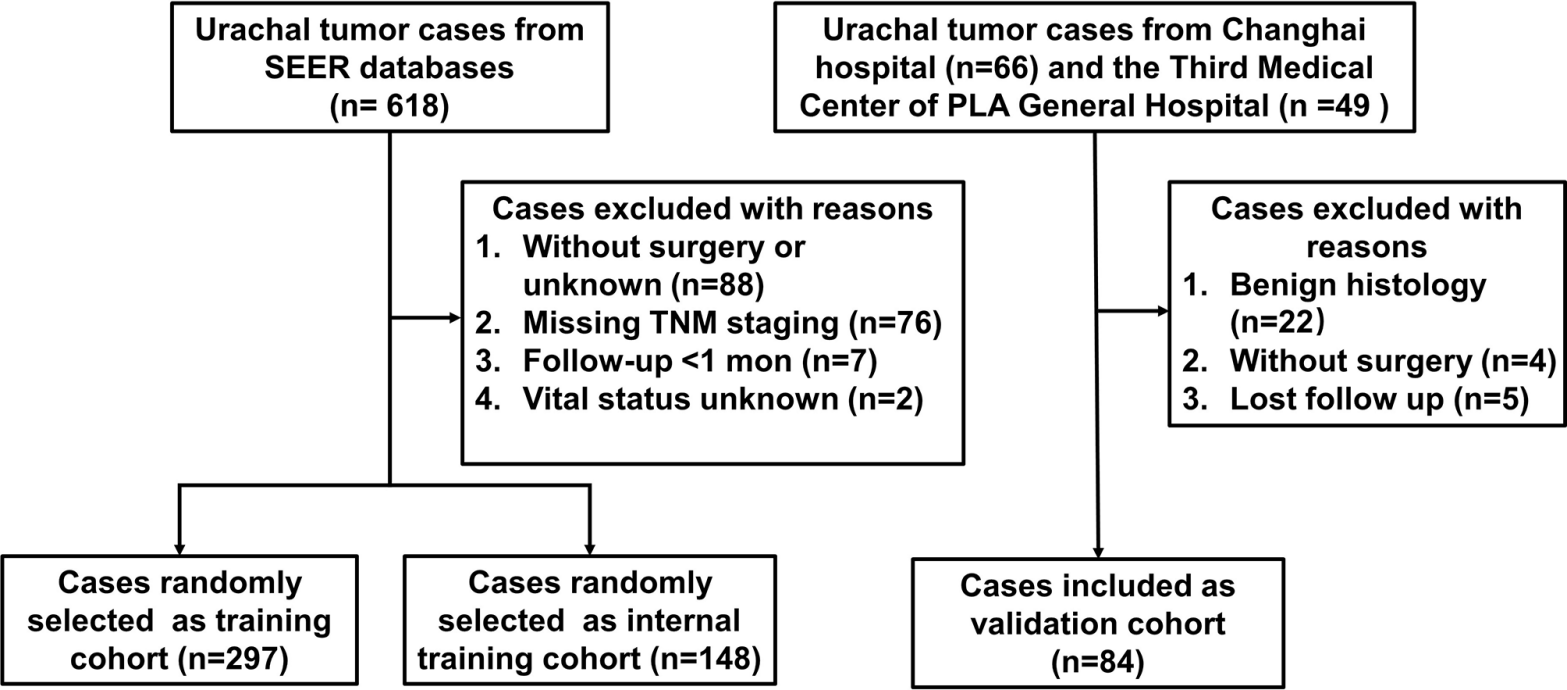
Flowchart illustrating patient selection for this study.

As shown in **Figure 1**, 445 patients, with median follow-up of 42 months, meeting the inclusion criteria were identified from SEER database (297 patients were randomly selected as training set, and 148 patients were selected as internal validation set). Meanwhile, 84 patients, with median follow-up of 34.5 months, were identified from two independent hospitals as external validation cohort. The demographic and clinicopathological variables of these two cohorts are listed in **Table 1**. Significant difference existed between the baseline characteristics of SEER cohort and external validation cohort. The 3- and 5-year CSS was 74.9% [95% confidence interval (CI), 69.8%–80.4%] and 64.6% (95% CI, 58.7%–71.0%) for SEER cohort, respectively. In the external validation cohort, the 3- and 5-year CSS was 65.9% (95% CI, 55.5%–78.2%) and 51.1% (95% CI, 39.9%–65.4%), respectively.

**Table 1:** Patient characteristics of training and validation cohort.

Variables	SEER Cohort (n = 445)	External Validation Cohort (n = 84)	*p*-value
**Age, median (IQR)**	58.0 (47 to 68)	52.5 (46–63)	0.11
**Gender, male/female**	241/204	58/26	0.01
**Median follow-up (IQR)**	42.0 (16 to 93)	34.5 (18.9–61.8)	<0.01
**Race (n%)**			<0.01
**White**	344 (77.3%)	–	
**Black**	46 (10.3%)	–	
**Asian or other**	55 (12.4%)	84 (100%)	
**Tumor size (cm)**			<0.01
**median (IQR)**	4.2 (3.0 to 7.0)	3.5 (2.5–5.0)	
**NA (n%)**	120 (27.0%)	3 (3.6%)	
**Surgery (n%)**			0.16
**TURBT**	48 (10.8%)	4 (4.8%)	
**Partial cystectomy**	343 (77.1%)	71 (84.5%)	
**Radical cystectomy**	54 (12.1%)	9 (10.7%)	
**Lymphadenectomy (n%)**			<0.01
**No or unknown**	242 (54.4%)	29 (34.5%)	
**Yes**	203 (45.6%)	55 (65.5%)	
**Grade (n%)**			<0.01
**Well to moderately differentiated (GI–II)**	229 (51.5%)	30 (35.7%)	
**Poorly differentiated or Undifferentiated (GIII–IV)**	126 (28.3%)	48 (57.1%))	
**Unknown**	90 (20.2%)	6 (7.1%)	
**Histology (n%)**			0.01
**Urothelial carcinoma**	38 (8.5%)	3 (3.6%)	
**Adenocarcinoma**	390 (87.6%)	81 (96.4%)	
**Other malignant type**	17 (3.8%)	0	
**T stage (n%)**			<0.01
**Ta-1**	79 (17.8%)	0	
**T2**	101 (22.7%)	5 (6.0%)	
**T3**	189 (42.5%)	63 (75.0%)	
**T4**	48 (10.8%)	11 (13.1%)	
**Tx**	28 (6.3%)	5 (6.0%)	
**Lymph node (n%)**			0.28
**Negative**	377 (84.7%)	66 (78.6%)	
**Positive**	34 (7.6%)	11 (13.1%)	
**Nx**	34 (7.6%)	7 (8.3%)	
**Metastasis (n%)**			0.15
**No**	382 (85.8%)	78 (92.9%)	
**Yes**	61 (13.7%)	6 (7.1%)	
**Mx**	2 (0.4%)	0	
**Sheldon stage (n%)**			<0.01
**I**	86 (19.3%)	0	
**II**	98 (22.0%)	6 (7.1%)	
**III**	156 (35.1%)	65 (77.4%)	
**IV**	105 (23.6%)	13 (15.5%)	
**Mayo stage (n%)**			<0.01
**I**	174 (39.1%)	5 (6.0%)	
**II**	166 (37.3%)	62 (73.8%)	
**III**	39 (8.8%)	13 (15.5%)	
**IV**	66 (14.8%)	4 (4.8%)	

IQR, interquartile range; SD, standard deviation; NA, not available.

### Development of Nomograms

**Figure 2:**
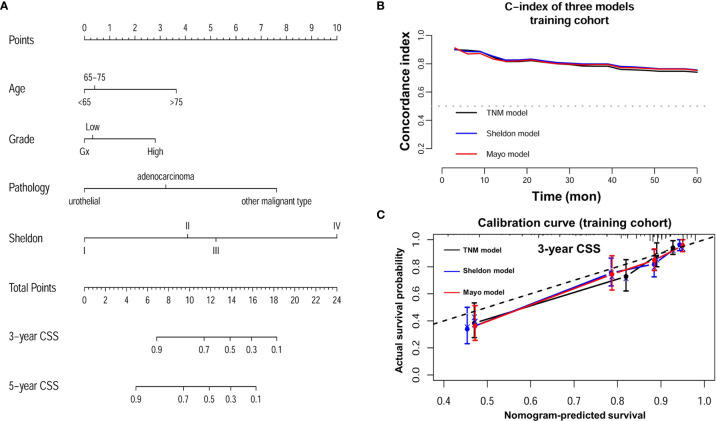
**(A)** Nomogram of Sheldon model for prediction of cancer specific survival (CSS) of urachal cancer. **(B)** C-index of three nomograms at different time points in the training cohort. **(C)** Calibration plot of three nomograms for prediction of CSS at 3 years in the training cohort.

Detailed results of univariable and multivariable Cox regression analysis of predicative variables from the training cohort are summarized in **Supplementary Tables S1** and **S2**. We established three nomograms (TNM, Sheldon, and Mayo models) based on the independent risk factors resulting from the multivariable Cox regression analysis (**Figure 2** and **Supplementary Figure S1**). The mean C-index of these models are listed in **Table 2** and **Figure 2**; the Sheldon model yielded the highest C-index. The calibration curves for CSS showed fair agreements between the prediction and actual observation in the training cohort (**Figure 2**).

**Table 2:** C-index of different nomogram models.

	Training cohort		Internal validation cohort		External validation cohort	
Models	Mean c-index	95%CI	Mean c-index	95%CI	Mean c-index	95%CI
**TNM**	**0.767**	**0.718–0.814**	**0.769**	**0.757–0.781**	**0.626**	**0.614–0.638**
**Sheldon**	**0.774**	**0.730–0.821**	**0.778**	**0.767–0.789**	**0.809**	**0.796–0.823**
**Mayo**	**0.763**	**0.714–0.812**	**0.743**	**0.731–0.755**	**0.776**	**0.759–0.793**

### Validation of the Nomograms and Risk Stratification

**Figure 3:**
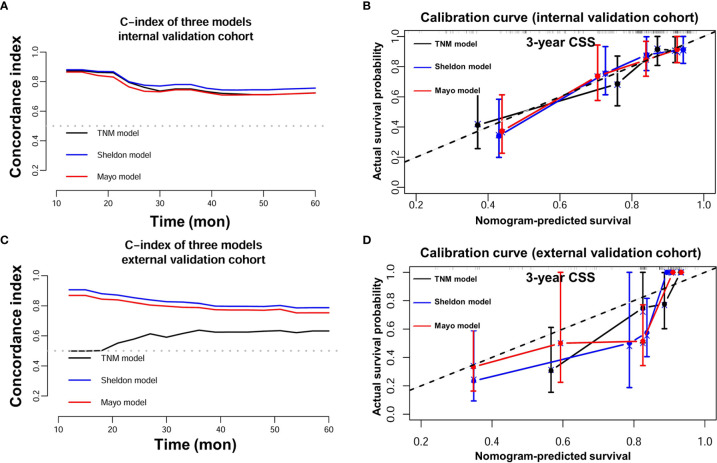
**(A)** C-index of three nomograms in the internal validation cohort. **(B)** Calibration plot of three nomograms in the internal validation cohort. **(C)** C-index of three nomograms in the external validation cohort. **(D)** Calibration plot of three nomograms in the external validation cohort.

**Figure 4:**
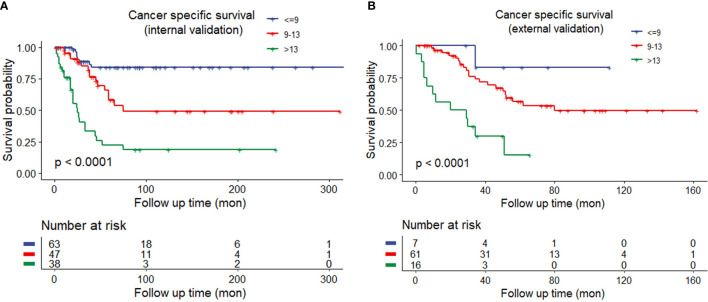
**(A)** Kaplan–Meier curves of different risk groups stratified by the Sheldon model in the internal training cohort. **(B)** Different risk groups stratified by the Sheldon model in the external validation cohort.

**Figure 5:**
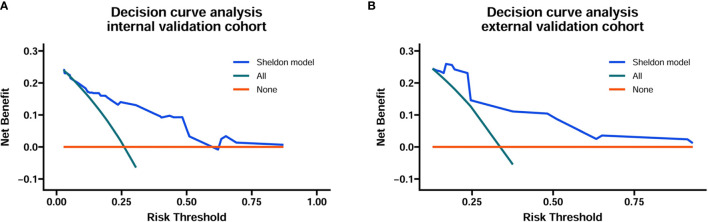
Decision curve analysis for the Sheldon model in the internal **(A)** and external **(B)** training cohort. The horizontal solid orange line represents the assumption that no patients will die, and the solid green line represents the assumption that all patients will die. On decision curve analyses, the nomogram showed superior net benefit across all range of threshold probabilities.

The discrimination ability of these nomograms was further validated in the internal validation and external validation datasets; the Sheldon model also had higher C-index than other two models, especially in the external validation cohort (**Table 2** and **Figure 2**). The calibration curve showed fair consistency between actual survival probability and the nomogram-predicted probability in the internal and external validation datasets (**Figure 3**). Since Sheldon staging is commonly used in clinical practice and the Sheldon model had superior discrimination power in predicting CSS compared to TNM and Mayo models, the Sheldon model was chosen as the optimal for further analysis. The X-title plots demonstrated that the optimal nomogram scores cutoff to classify low-, middle-, and high-risk groups were 9 and 13 points (**Supplementary Figure S2**). Kaplan–Meier curves for CSS outcomes of the different risk subgroups revealed significant distinction in survival probability both in the internal and external validation cohort (**Figure 4**). DCA analysis was conducted to illustrate the net benefit of Sheldon model in the internal and external validation datasets, which demonstrated that the use of the nomogram provided greater net benefit to predict the prognosis at all different threshold (**Figure 5**).

## Discussion

Urachal cancer is referred as a highly malignant cancer and remains understudied due to its rarity, which accounts for 0.2%–0.7% of all bladder tumors and approximately 10–30% of all adenocarcinomas of the bladder (18). The median age of diagnosis for urachal cancer is approximately 50–60 years, which is on average 10 years earlier than bladder cancer (6). Although urachal cancer is usually located at the dome of bladder, the most common histological type is adenocarcinomas, and they do not behave as typical adenocarcinoma or urothelial carcinoma (3, 19). The molecular profile studies have revealed that urachal cancer has closer resemblance to colorectal than to bladder cancer on the genomic level (20). To date, the most commonly recommended treatment for non-metastatic urachal cancer is partial cystectomy, while there is no consensus on whether neo-adjuvant, adjuvant chemotherapy, or radiation could be beneficial in improving survival. It is suggested that treating urachal patients at high risk of relapse with adjuvant chemotherapy seems reasonable (8). As a result, a risk stratification model for urachal cancer is needed because improved prediction of postoperative prognosis could be helpful to guide patient counseling, administration of adjuvant therapies, surveillance scheduling, and design of clinical trials (21).

Currently, few studies have investigated prognostic factors that will allow clinicians to predict prognosis of urachal cancer. Sheldon staging system, which was proposed in 1984 for urachal cancer, remains the most commonly used in clinical practice, while TNM system for bladder cancer and Mayo system were less frequently used (4). Szarvas et al. (5) suggested that TNM staging is limited, since urachal cancer did not originate from the urothelium of bladder. However, there were studies that proved TNM staging to be a main predictor of prognosis for urachal cancer (22). When using the Sheldon staging system, most patients are classified into stage III and few patients into other stages. In contrast, Mayo staging system provides a more balanced distribution of patients between stages (5). Dhillon et al. (22) demonstrated that both staging systems could be able to significantly predict patients’ prognosis. Kim et al. (23) suggested that the Mayo staging might be more effective and simpler than the Sheldon staging system. Here, we also revealed that Sheldon and Mayo staging system both were main independent predictors of prognosis. We found that patients with positive lymph nodes (Mayo staging III) or metastasis (Mayo staging IV) had similar poor CSS, which indicated that these patients could be categorized as one group like Sheldon staging IV.

In the present study, we developed nomograms with independent risk factors obtained from readily available clinicopathological variables. Nomograms including three different staging systems, namely, TNM, Sheldon, and Mayo staging system, were established. Except for Sheldon stage, this study further suggested that older age, higher tumor grade, and other malignant histological type rather than urothelial carcinoma and adenocarcinoma were independent risk factors for poorer prognosis. The respective c-index of nomogram with Sheldon staging was 0.78 and 0.81 in the internal and external validation cohort, both of which were higher than the other two nomogram models. We further defined the cutoff threshold of the total scores obtained from the nomogram that could help to divide patients into different risk groups with distinct prognosis. The present risk stratification model may help clinicians to predict postoperative prognosis and determine surveillance strategies and adjuvant therapies. As for patients in the high-risk group, adjuvant treatment regimens could be considered to improve the prognosis. So far, adjuvant chemotherapies, including cisplatin-based treatment modalities and 5-fluorouracil containing regimes, have shown partial regression of tumor burden in small cohorts for urachal cancer of advanced stage (3, 5, 24). Consistent with a previous study by Duan et al. (2), we failed to find that lymphadenectomy was an independent prognostic factor for CSS; however, lymphadenectomy has important role in accurate staging, which is essential for predicting prognosis.

Due to the clinical and genetic similarities between urachal cancer and gastroenteric cancer, studies have explored the predicative value of serum markers like CEA, CA724, CA19-9, and CA125 in urachal cancer (4, 25, 26). Although studies showed these serum markers had little value in predicting prognosis, they might be useful for monitoring response to therapy during follow-up (4). Several studies have investigated the predicative role of immunohistochemical markers for urachal cancer; however, these markers like CEA, CK7, CK 20, Ki-67, P53, P21, and P27 were not correlated with prognosis (27–29). Módos et al. (30) analyzed the prognostic role of genetic mutations in urachal cancer; they found frequent mutations, such as KRAS, BRAF, NRAS, EGFR, and PIK3CA, were not correlated with prognosis. So far, serum, immunohistochemical, and genetic markers showed little potential for risk classification; future studies with collaboration of multiple centers and larger cohort may be necessary to find potential predicative markers for urachal cancer.

There are some major limitations that should be noted in the present study. First, although this study collected a relatively large cohort from SEER database, this registry-based retrospective study and its intrinsic biases must be acknowledged. The information of several important clinical variables, such as tumor size and surgical margin, were incomplete or not available. The adjuvant treatment strategies of training cohort were not available from the SEER database, even though there are no firm evidence that palliative chemotherapy confers survival benefit for recurrent and metastatic urachal cancer (7). Second, the Sheldon and Mayo staging for training cohort may not be accurate because they were not readily available from the SEER database; we reclassified these two staging classifications based on available TNM staging and registered clinical information. Even so, our external validation found that the nomogram containing Sheldon and Mayo staging system developed from the training cohort had a high c-index, which revealed its value for clinical application. Third, serum, immunohistochemical, and genetic markers were not available for analysis in the SEER database; further prediction models including potential predicative markers for urachal cancer may be helpful.

## Conclusions

The present study developed and externally validated three novel and accurate nomograms for predicting the CSS of urachal cancer using readily available clinicopathological variables. The nomogram with Sheldon staging system was recommended because of its better predicative value and simplicity to use. The proposed nomograms have promising clinical applicability to help clinicians on individualized patient counseling, decision-making, and clinical trial designing.

## Data Availability Statement

The original contributions presented in the study are included in the article/**Supplementary Material**. Further inquiries can be directed to the corresponding authors.

## Ethics Statement

The studies involving human participants were reviewed and approved by the Ethical Boards of Changhai Hospital and the Third Medical Center of PLA General Hospital. The patients/participants provided their written informed consent to participate in this study.

## Author Contributions

SZ and CX were responsible for the study design and participated in the evaluation of results. XY, CM, and MW participated in the collection of study materials or patients. YY, XA, and ZZ participated in the collection and assembly of data. SZ and XY conducted the data analysis and interpretation. SZ, XY, CM, and MW drafted the manuscript. CX and LW proofread the manuscript for important intellectual content. All authors contributed to the article and approved the submitted version.

## Funding

This research was financed by grants from the Qihang program of Naval Medical University, Shanghai Sailing Program (18YF1422700), Shanghai Pujiang Program (18PJD058), and the National Natural Science Foundation of China (81772720, 81572509, 81802515, 81801854, and 82172871).

## Conflict of Interest

The authors declare that the research was conducted in the absence of any commercial or financial relationships that could be construed as a potential conflict of interest.

## Publisher’s Note

All claims expressed in this article are solely those of the authors and do not necessarily represent those of their affiliated organizations, or those of the publisher, the editors and the reviewers. Any product that may be evaluated in this article, or claim that may be made by its manufacturer, is not guaranteed or endorsed by the publisher.

## Supplementary Material

Supplementary Figure 1 **(A)** Nomogram of TNM model for prediction of cancer specific survival of urachal cancer; **(B)** Nomogram of Mayo model for prediction of cancer specific survival of urachal cancer.

Supplementary Figure 2 **(A)** X-tile plots to identify the optimal risk score cutoff based on cancer specific survival in the SEER training set. The X-tile plot was generated by dividing risk scores into three populations (low, middle and high). Each pixel (point) of the X-tile plot represents the data from a given set of divisions. The X-axis represents all potential risk score cutoff from low to high (left to right) that define low subset, whereas the Y-axis represents risk score cutoff value from high to low (top to bottom), that define a high subset. The arrows represent the direction in which the low subset (X-axis) and the high subset (Y-axis) increase in size. The coloration of the plot represents the strength of the association at each division, ranging from low (dark, black) to high (bright, red or green). Inverse associations between the risk score and survival are colored red, whereas direct associations are colored green. The present figure suggested higher risk score correlated with worse prognosis. **(B)** The distributions of the number of patients by risk score, 9 and 13 was the optimal cutoff value to define low-, middle- and high-risk of urachal cancer. **(C)** Kaplan-Meier curves of different risk groups stratified by the Sheldon model in the training cohort.
