# Supplementary figures and images for: Construction and Validation of Novel Prediction Tools Based on Large Population-Based Database to Predict the Prognosis of Urachal Cancer After Surgery
Source: Front Oncol. 2021 Sep 14;11:718691. doi: 10.3389/fonc.2021.718691 (PMC8476958; doi:10.3389/fonc.2021.718691)

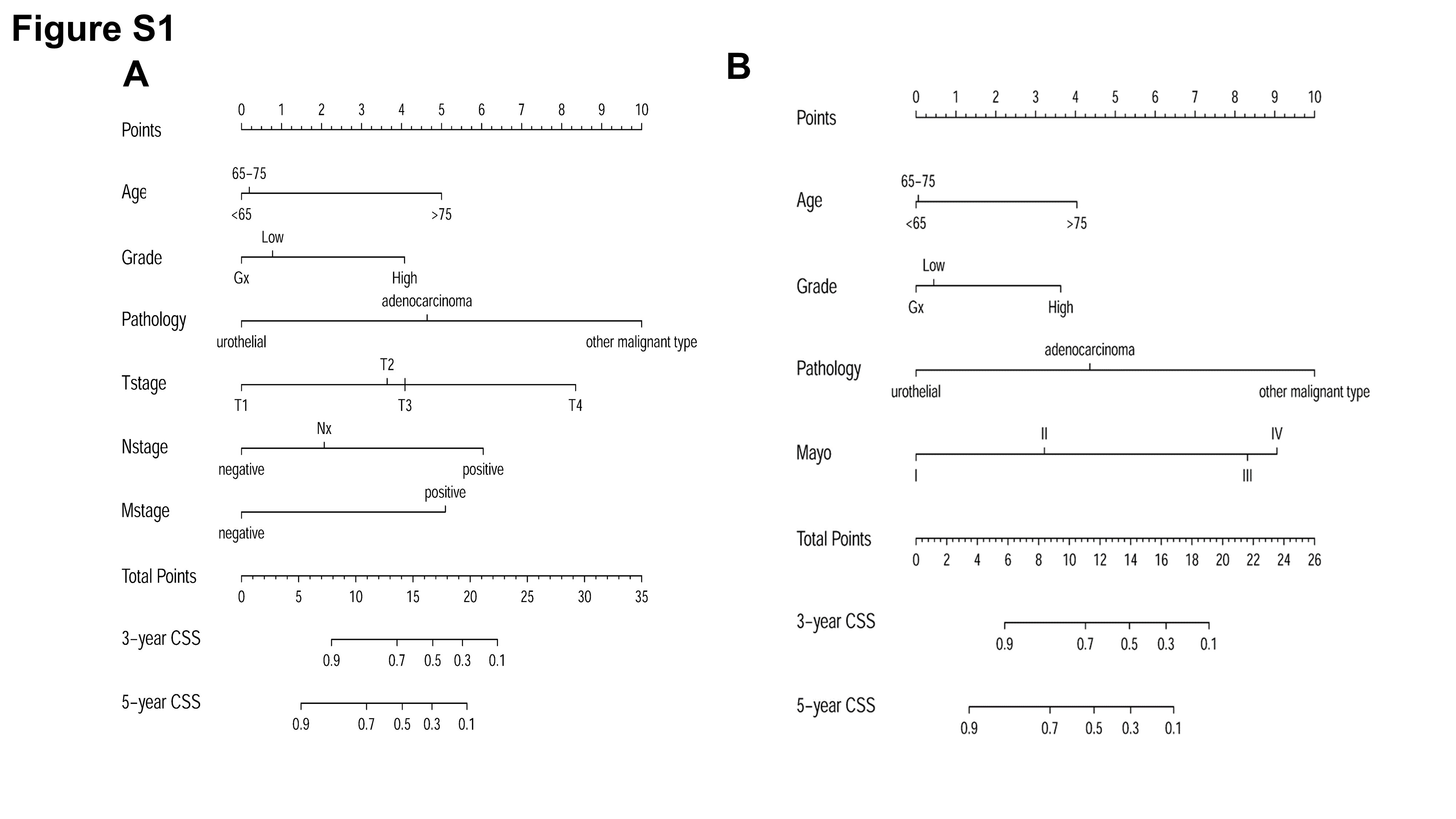

Supplement: Supplementary Figure 1 — (A) Nomogram of TNM model for prediction of cancer specific survival of urachal cancer; (B) Nomogram of Mayo model for prediction of cancer specific survival of urachal cancer. [file Image_1.jpeg]

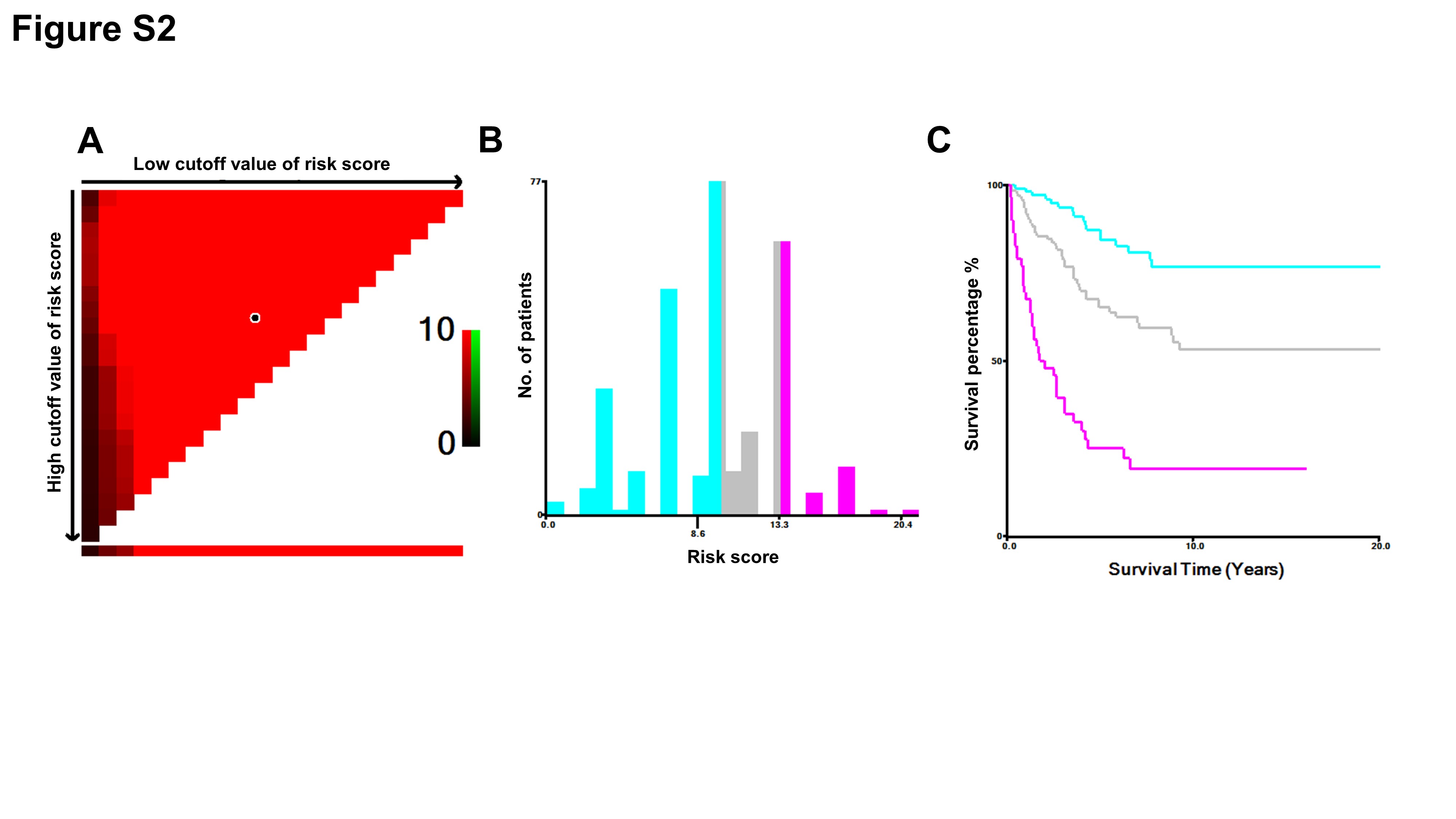

Supplement: Supplementary Figure 2 — (A) X-tile plots to identify the optimal risk score cutoff based on cancer specific survival in the SEER training set. The X-tile plot was generated by dividing risk scores into three populations (low, middle and high). Each pixel (point) of the X-tile plot represents the data from a given set of divisions. The X-axis represents all potential risk score cutoff from low to high (left to right) that define low subset, whereas the Y-axis represents risk score cutoff value from high to low (top to bottom), that define a high subset. The arrows represent the direction in which the low subset (X-axis) and the high subset (Y-axis) increase in size. The coloration of the plot represents the strength of the association at each division, ranging from low (dark, black) to high (bright, red or green). Inverse associations between the risk score and survival are colored red, whereas direct associations are colored green. The present figure suggested higher risk score correlated with worse prognosis. (B) The distributions of the number of patients by risk score, 9 and 13 was the optimal cutoff value to define low-, middle- and high-risk of urachal cancer. (C) Kaplan-Meier curves of different risk groups stratified by the Sheldon model in the training cohort. [file Image_2.jpeg]
