# Supplementary material for: Construction and Validation of Novel Prediction Tools Based on Large Population-Based Database to Predict the Prognosis of Urachal Cancer After Surgery
Source: Front Oncol. 2021 Sep 14;11:718691. doi: 10.3389/fonc.2021.718691 (PMC8476958; doi:10.3389/fonc.2021.718691)

Table S1. Univariable Cox regression analysis of training cohort for predicting cancer specific survival

Variables	HR	95% CI	P value
**Age, median (<= 65 as referent)**			**0.04**
65 to 75 yrs	0.8	0.51−1.26	0.34
>75 yrs	1.68	1.04−2.72	0.03
**Gender (female as referent)**			
Male	0.73	0.53−1.00	0.05
**Race (white as referent)**			**0.19**
Black	1.54	0.96−2.49	0.07
Asian or other	1.19	0.72−1.95	0.5
**Surgery (TURBT as referent)**			**<0.01**
Partial cystectomy	0.57	0.36−0.91	0.02
Radical cystectomy	1	0.56−1.79	0.99
**Lymphadenectomy (no as referent)**			
Yes	0.95	0.69−1.32	0.77
**Grade (well to moderate differentiated as referent)**			**0.03**
poor or undifferentiated	2.52	1.77−3.60	<0.01
Gx	1.03	0.65−1.63	0.89
**Histology (Urothelial carcinoma as referent)**			**0.15**
Adenocarcinoma	2.46	1.08−5.56	0.03
Other malignant type	6.24	2.27−17.17	<0.01
**T stage (Ta−1 as referent)**			**<0.01**
T2	3.05	1.43−6.51	0.09
T3	4.43	2.21−8.87	<0.01
T4	10.09	4.73−21.51	<0.01
Tx	7.6	3.36−17.2	<0.01
**Lymph node (negative as referent)**			**<0.01**
Positive	5.12	3.21−8.17	<0.01
Nx	2.73	1.66−4.50	<0.01
**Metastasis (negative as referent)**			
Positive	4.63	3.19−6.72	<0.01
Mx	5.55	1.36−22.68	0.01
**Sheldon Stage (I as referent)**			**<0.01**
II	3.84	1.64−9.00	<0.01
III	5.59	2.55−12.27	<0.01
IV	16.77	7.66−36.72	<0.01
**Mayo stage (I as referent)**			**<0.01**
II	2.43	1.54−3.81	<0.01
III	6.22	3.57−10.82	<0.01
IV	8.27	5.08−13.44	<0.01

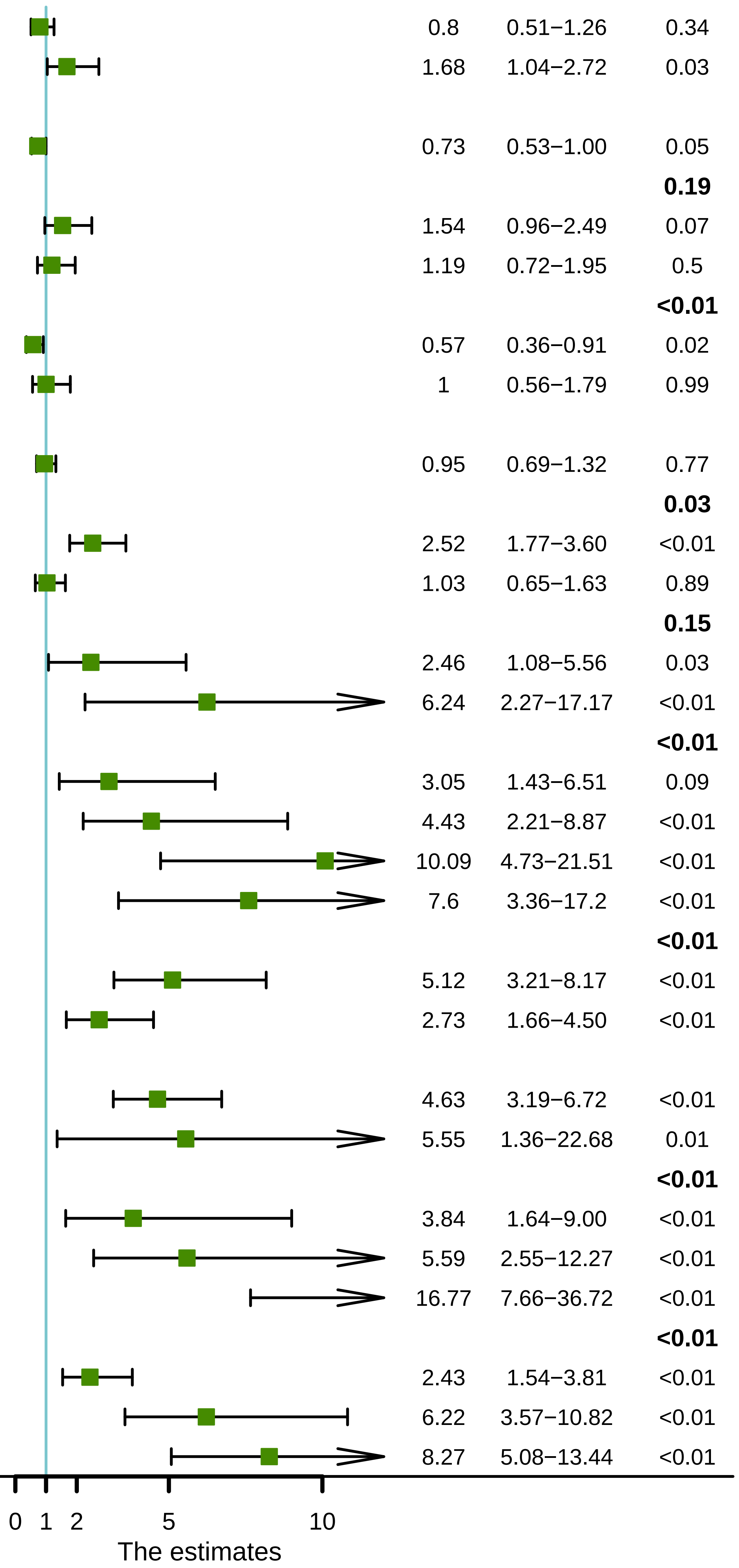

Supplement: Supplementary file 3 [file Table_1.pdf]
