# Supplementary material for: Construction and Validation of Novel Prediction Tools Based on Large Population-Based Database to Predict the Prognosis of Urachal Cancer After Surgery
Source: Front Oncol. 2021 Sep 14;11:718691. doi: 10.3389/fonc.2021.718691 (PMC8476958; doi:10.3389/fonc.2021.718691)

Table S2. Multivariable Cox regression analysis of training cohort for predicting cancer specific survival

Variables	HR	95% CI	P value
**TNM model**			
**Age, median (<= 65 as referent)**			**<0.01**
65 to 75 yrs	1.03	0.64−1.65	0.84
>75 yrs	2.85	1.70−4.74	<0.01
**Histology (Urothelial carcinoma as referent)**			**<0.01**
Adenocarcinoma	2.65	1.08−6.51	0.03
Other malignant type	7.29	2.29−23.20	<0.01
**Grade (well to moderate differentiated as referent)**			**<0.01**
poor or undifferentiated	1.96	1.35−2.85	<0.01
Gx	0.84	0.52−1.35	0.47
**T stage (Ta−1 as referent)**			**<0.01**
T2	2.18	1.00−4.78	0.05
T3	2.41	1.16−5.04	0.02
T4	5.89	2.65−13.09	<0.01
Tx	2.52	0.99−6.37	0.05
**Lymph node (negative as referent)**			**<0.01**
Positive	3.53	2.09−5.97	<0.01
Nx	1.40	0.76−2.60	0.28
**Metastasis (negative as referent)**			**<0.01**
Positive	2.96	1.88−4.68	<0.01
Mx	1.84	0.35−9.57	0.47
**Sheldon model**			
**Age, median (<= 65 as referent)**			**<0.01**
65 to 75 yrs	1.11	0.69−1.79	0.67
>75 yrs	2.63	1.61−4.30	<0.01
**Histology (Urothelial carcinoma as referent)**			**<0.01**
Adenocarcinoma	2.36	0.96−5.78	0.06
Other malignant type	7.55	2.50−22.77	<0.01
**Grade (well to moderate differentiated as referent)**			**<0.01**
poor or undifferentiated	1.95	1.35−2.82	<0.01
Gx	0.92	0.58−1.47	0.72
**Sheldon Stage (I as referent)**			**<0.01**
II	3.02	1.26−7.20	0.01
III	4.08	1.80−9.24	<0.01
IV	14.68	6.55−32.88	<0.01
**Mayo model**			
**Age, median (<= 65 as referent)**			**<0.01**
65 to 75 yrs	1.01	0.63−1.62	0.98
>75 yrs	2.50	1.52−4.12	<0.01
**Histology (Urothelial carcinoma as referent)**			**<0.01**
Adenocarcinoma	2.71	1.13−6.54	0.03
Other malignant type	9.76	3.32−28.64	<0.01
**Grade (well to moderate differentiated as referent)**			**<0.01**
poor or undifferentiated	2.09	1.45−3.00	<0.01
Gx	0.91	0.57−1.44	0.68
**Mayo stage (I as referent)**			**<0.01**
II	2.10	1.31−3.35	<0.01
III	6.73	3.80−11.90	<0.01
IV	8.00	4.83−13.23	<0.01

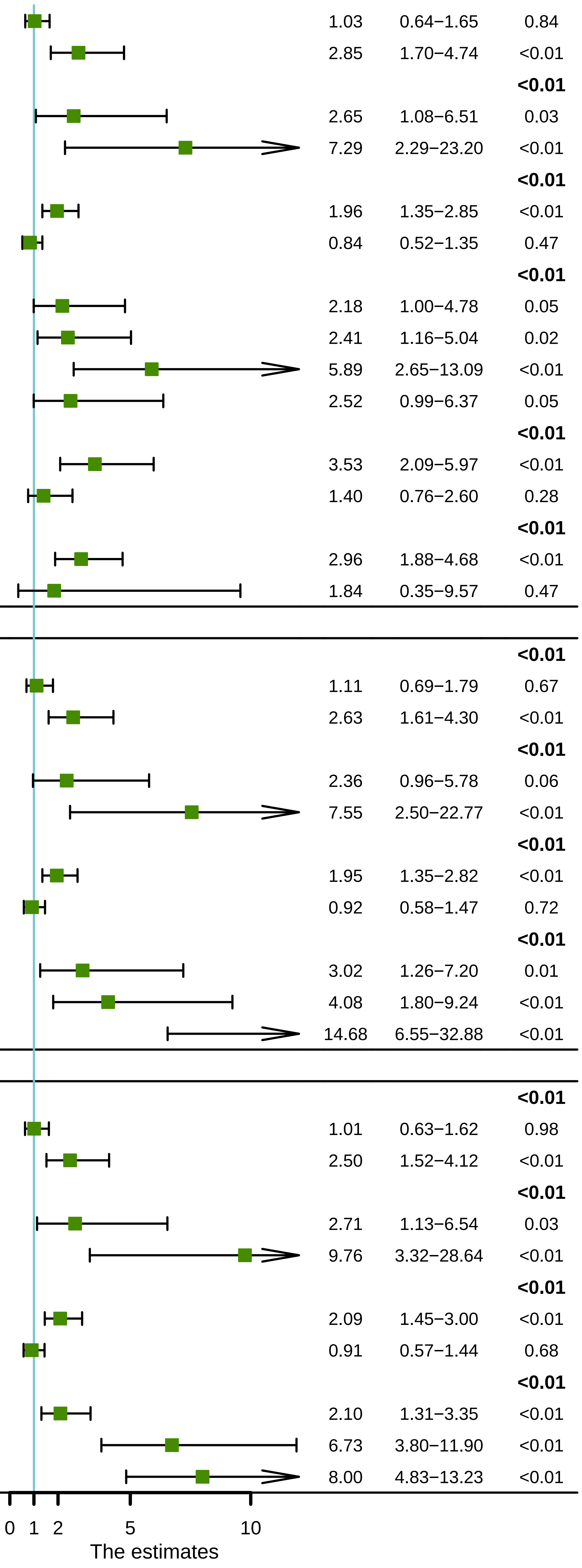

Supplement: Supplementary file 4 [file Table_2.pdf]
